# Multiparametric magnetic resonance imaging of experimental chronic kidney disease: A quantitative correlation study with histology

**DOI:** 10.1371/journal.pone.0200259

**Published:** 2018-07-16

**Authors:** Gunnar Schley, Jutta Jordan, Stephan Ellmann, Seymour Rosen, Kai-Uwe Eckardt, Michael Uder, Carsten Willam, Tobias Bäuerle

**Affiliations:** 1 Department of Nephrology and Hypertension, Friedrich-Alexander University Erlangen-Nürnberg (FAU) and University Hospital Erlangen, Erlangen, Germany; 2 Department of Radiology, Friedrich-Alexander University Erlangen-Nürnberg (FAU) and University Hospital Erlangen, Erlangen, Germany; 3 Department of Pathology, Beth Israel Deaconess Medical Center, Boston, Massachusetts, United States of America; 4 Department of Nephrology and Medical Intensive Care, Charité –Universitätsmedizin Berlin, Berlin, Germany; Baker IDI Heart and Diabetes Institute, AUSTRALIA

## Abstract

**Objectives:**

In human chronic kidney disease (CKD) the extent of renal tubulointerstitial fibrosis correlates with progressive loss of renal function. However, fibrosis can so far only be assessed by histology of kidney biopsies. Magnetic resonance imaging (MRI) can provide information about tissue architecture, but its potential to assess fibrosis and inflammation in diseased kidneys remains poorly defined.

**Materials and methods:**

We evaluated excised kidneys in a murine adenine-induced nephropathy model for CKD by MRI and correlated quantitative MRI parameters (T1, T2, and T2* relaxation times, apparent diffusion coefficient and fractional anisotropy) with histological hallmarks of progressive CKD, including renal fibrosis, inflammation, and microvascular rarefaction. Furthermore, we analyzed the effects of paraformaldehyde fixation on MRI parameters by comparing kidney samples before and after fixation with paraformaldehyde.

**Results:**

In diseased kidneys T2 and T2* relaxation times, apparent diffusion coefficient and fractional anisotropy in the renal cortex and/or outer medulla were significantly different from those in control kidneys. In particular, T2 relaxation time was the best parameter to distinguish control and CKD groups and correlated very well with the extent of fibrosis, inflammatory infiltrates, tubular dilation, crystal deposition, and loss of peritubular capillaries and normal tubules in the renal cortex and outer medulla. Fixation with paraformaldehyde had no impact on T2 relaxation time and fractional anisotropy, whereas T1 times significantly decreased and T2* times and apparent diffusion coefficients increased in fixed kidney tissue.

**Conclusions:**

MRI parameters provide a promising approach to quantitatively assess renal fibrosis and inflammation in CKD. Especially T2 relaxation time correlates well with histological features of CKD and is not influenced by paraformaldehyde fixation of kidney samples. Thus, T2 relaxation time might be a candidate parameter for non-invasive assessment of renal fibrosis in human patients.

## Introduction

Chronic kidney disease (CKD) is considered a major health problem affecting approximately 14% of the general adult population in the United States [1]. It is associated with high morbidity, all-cause and cardiovascular mortality, and health care expenditures [2]. CKD can progress to end-stage renal disease requiring dialysis or kidney transplantation [3]. It is defined as the presence of persistent (>3 months) structural or functional kidney abnormalities (detected by urinalysis, imaging studies, or histology) with or without decreased glomerular filtration rate (GFR) (<60 ml/min/1.73 m^2^) [4]. Irrespective of the underlying cause, the final common manifestation of progressive kidney diseases is glomerulosclerosis and tubulointerstitial fibrosis involving replacement of normal renal parenchyma by non-functional tissue [5,6]. Even in glomerular diseases tubulointerstitial changes have been shown to be the best predictors of deteriorating renal function and disease progression [7,8]. Thus, assessing the degree of renal fibrosis has diagnostic, prognostic, and potential therapeutic implications. At present renal fibrosis is determined by tissue histology of kidney biopsies. However, kidney biopsies are invasive and associated with possible complications. Moreover, sampling errors due to the focal nature of renal fibrosis may lead to misinterpretation [9]. Quantitative magnetic resonance imaging (MRI) approaches, such as mapping techniques and diffusion weighted imaging, could potentially discriminate histological changes in kidneys non-invasively [10–13]. However, until now non-contrast enhanced quantitative MRI parameters for the assessment of histopathological changes in renal tissue have been evaluated in only few experimental studies [14–18]. In mouse models of acute kidney injury (AKI) and kidney transplantation, quantitative MRI using multiple parameters not depending on the application of contrast agents identified disease-specific renal changes. T1 and T2 relaxation times as well as the apparent diffusion coefficient (ADC) were associated with severity of acute renal changes and kidney volume loss after AKI as well as acute renal allograft rejection [14–18]. To the best of our knowledge in CKD models comprehensive multiparametric MRI has not yet been investigated in quantitative correlation with histology. Therefore, the aim of our study was to find suitable MRI parameters reflecting typical histopathological changes in CKD in contrast to healthy control kidneys. We examined quantitative MRI parameters in murine kidneys with chronic tubulointerstitial nephritis by correlating imaging results with histopathological analyses.

## Materials and methods

### Animal experiments

All animal experiments were approved by the animal care and use committee of local government authorities (Regierung von Mittelfranken, Ansbach, Germany; Az 54–2532.1-11/13) and conducted in strict accordance with the Guide for the Care and Use of Laboratory Animals of the National Institutes of Health. Twenty-four male C57BL/6NCrl mice, aged between 12 and 15 weeks weighing 25 to 30 g, were obtained from Charles River (Sulzfeld, Germany) and acclimated for at least 7 days prior to study initiation. They were housed in groups of four animals per cage under a 12:12 hour light-dark cycle at constant temperature (22±1°C) and humidity (55^2^5%) with free access to food and tap water. Mice were randomly assigned into two groups, including the control (Ctrl) group (n = 12) fed with standard rodent chow (V1534-000, ssniff Spezialdiäten, Soest, Germany) and the CKD group (n = 12) receiving a diet supplemented with 0.2% (w/w) adenine. Body weight and clinical status of mice were monitored daily. After 3 weeks mice were sacrificed by exsanguination under deep isoflurane anesthesia, and their kidneys were harvested. Kidneys from 8 control and 8 adenine-fed mice were fixed with paraformaldehyde for 16 hours before imaging and (immuno-) histological studies. Kidneys from 4 control and 4 CKD mice were subjected to MRI directly after removal and 16 hours after paraformaldehyde fixation.

### Histology and immunohistochemistry

Kidneys were fixed by immersion in 4% paraformaldehyde for 16 hours, washed in phosphate-buffered saline and embedded in paraffin as described previously [19]. Kidney sections (2 μm) were stained with hematoxylin and eosin (H&E) or Sirius red for the evaluation of renal histology and interstitial collagen deposition. Immunohistochemistry was performed with the following primary antibodies: rabbit polyclonal anti-mouse collagen IV (1:100; AbD Serotec 2150–1470, Kidlington, UK), rabbit polyclonal anti-mouse fibronectin (1:250; Lab Vision RB-077, Fremont, CA), rat monoclonal anti-mouse F4/80 (1:500; AbD Serotec MCA497GA) for macrophages, rat monoclonal anti-mouse NIMP-R14 (1:5,000; Abcam ab2557, Cambridge, UK) for neutrophils, and rat monoclonal anti-mouse MECA-32 (1:5; supernatant from hybridoma cell line) for endothelial cells. A biotinylated goat polyclonal anti-rat antibody (1:200 to 1:500; AbD Serotec STAR131B) and a biotinylated goat polyclonal anti-rabbit antibody (1:500; Vector Laboratories BA-1000, Burlingame, CA) were used as secondary antibodies and detected using the Vectastain Elite ABC Kit (PK-6100, Vector Laboratories) or the TSA Plus Biotin Kit (NEL749A001KT, Perkin Elmer, Waltham, MA) and the chromogen 3,3’-diaminobenzidine (K1500, Dako, Glostrup, Denmark). At least 10 non-overlapping digital microphotographs of cortex and outer medulla from each kidney slice were obtained separately at 5- to 20-fold magnification using a Leica DFC450 C digital camera mounted on a Leica DM6000 B microscope (Leica Microsystems, Wetzlar, Germany). The area stained positive for Sirius red, collagen IV, fibronectin, F4/80, NIMP-R14, and MECA-32 as well as the number of cell nuclei and birefringent dihydroxyadenine crystals (in polarized light) were quantified using ImageJ software version 1.51 [20]. Normal renal tubules were identified by their bright eosinophilic cytoplasm, and their mean area in control mice was set as 100%. The luminal area of renal tubules was evaluated as described previously [21].

### Magnetic resonance imaging

MRI was performed on a 7 Tesla small animal MRI scanner (ClinScan 70/30; Bruker, Ettlingen, Germany) using a whole-body mouse volume coil (Bruker). Kidneys (either unfixed or fixed with 4% paraformaldehyde for 16 hours) were embedded in agarose (1.5%) to avoid motion artefacts during MRI. The measurement protocol consisted of a T2 weighted Turbo-Spin-Echo (TSE) sequence for volumetric segmentation as well as sequences for assessment of relaxation times (T1, T2 and T2* times) and diffusion characteristic maps (apparent diffusion coefficient, ADC, and fractional anisotropy, FA) with the following parameters:

- T2 weighted Turbo-Spin-Echo (TSE) (time to repetition (TR): 3420 ms, time to echo (TE): 46 ms, flip angle: 140°, bandwidth (bw): 128 Hz/px, 32 averages, transversal base resolution matrix: 640 x 640),
- T1 relaxation map (3D Fast Low-Angle Shot (FLASH) sequence, TR: 50 ms, TE: 2.5 ms; flip angles: 8° and 42°, bw: 460 Hz/px, 3 averages, transversal base resolution matrix: 576 x 576),
- T2 relaxation map (fat saturated spin echo sequence, TR: 10 s, TEs in 18 interval steps of 10.2 ms starting from TE 10.2, flip angle: 180°, bw: 310 Hz/px, 2 averages, transversal base resolution matrix: 576 x 576),
- T2* relaxation map (gradient echo sequence, TR: 983 ms, 12 TEs in intervals from 3.98 ms, starting with 4 ms, flip angle: 40°, bw: 260 Hz/px, 3 averages, transversal base resolution matrix: 512 x 512),
- Diffusion-weighted imaging/diffusion tensor imaging (DTI) including apparent diffusion coefficient (ADC) and fractional anisotropy (FA) (single shot echo planar imaging sequence, 256 diffusion directions, 8 diffusion weighted b-values in steps of 200 s/mm^2^ ranging from b: 0–14000 s/mm^2^, TR: 8000 ms, TE: 60 ms, flip angle: 90°; bw: 1860 Hz/px, transversal base resolution matrix: 128 x 128).

The field of view was 30 mm x 30 mm for all sequences, and the slice thickness was 0.5 mm (except DTI, 1.0 mm). All maps were calculated automatically by Syngo software (Siemens, Erlangen, Germany).

### Image postprocessing

For image postprocessing and data analysis, a dedicated software package (Chimaera GmbH, Erlangen, Germany) was used to segment cortex, outer medulla and total kidney on the TSE sequence. Subsequently, the segmented volumes were transferred on the maps for determination of relaxation times (T1, T2, T2*) and diffusion properties (ADC, FA).

### Statistical analysis

Statistical analysis was performed using IBM SPSS Statistics for Windows version 21.0 (IBM, Armonk, NY) and GraphPad Prism version 5.04 for Windows (GraphPad Software, La Jolla, CA). All data are presented as mean ± standard error. Mann-Whitney U or Wilcoxon matched-pairs signed-rank tests were used for comparison of two independent groups. Statistical significance was defined as an explorative p value <0.05. Correlation of MRI and histological parameters was assessed using Pearson’s correlation coefficient *r*. The strength of associations was labeled as follows: for absolute values of *r*, 0–0.19 was regarded as very weak, 0.2–0.39 as weak, 0.4–0.59 as moderate, 0.6–0.79 as strong and 0.8–1 as very strong correlation [22]. Statistical significance of Pearson’s correlation coefficients was tested by two-tailed t-test.

## Results

### Comparison of MRI parameters between control and CKD mice

An adenine-enriched diet induces precipitation of 2,8-dihydroxyadenine crystals in the kidneys resulting in tubular atrophy, tubulointerstitial inflammation and fibrosis as well as progressive renal dysfunction resembling many characteristics of human CKD [23–25]. To acquire high resolution imaging data for subsequent quantitative correlation with histopathology, paraformaldehyde-fixed control and CKD kidneys were analyzed by *ex vivo* MRI. Relaxation times (T1, T2, and T2* time) and diffusion properties (ADC, FA) were determined in the renal cortex, outer medulla and total kidney volume.

**Fig 1:**
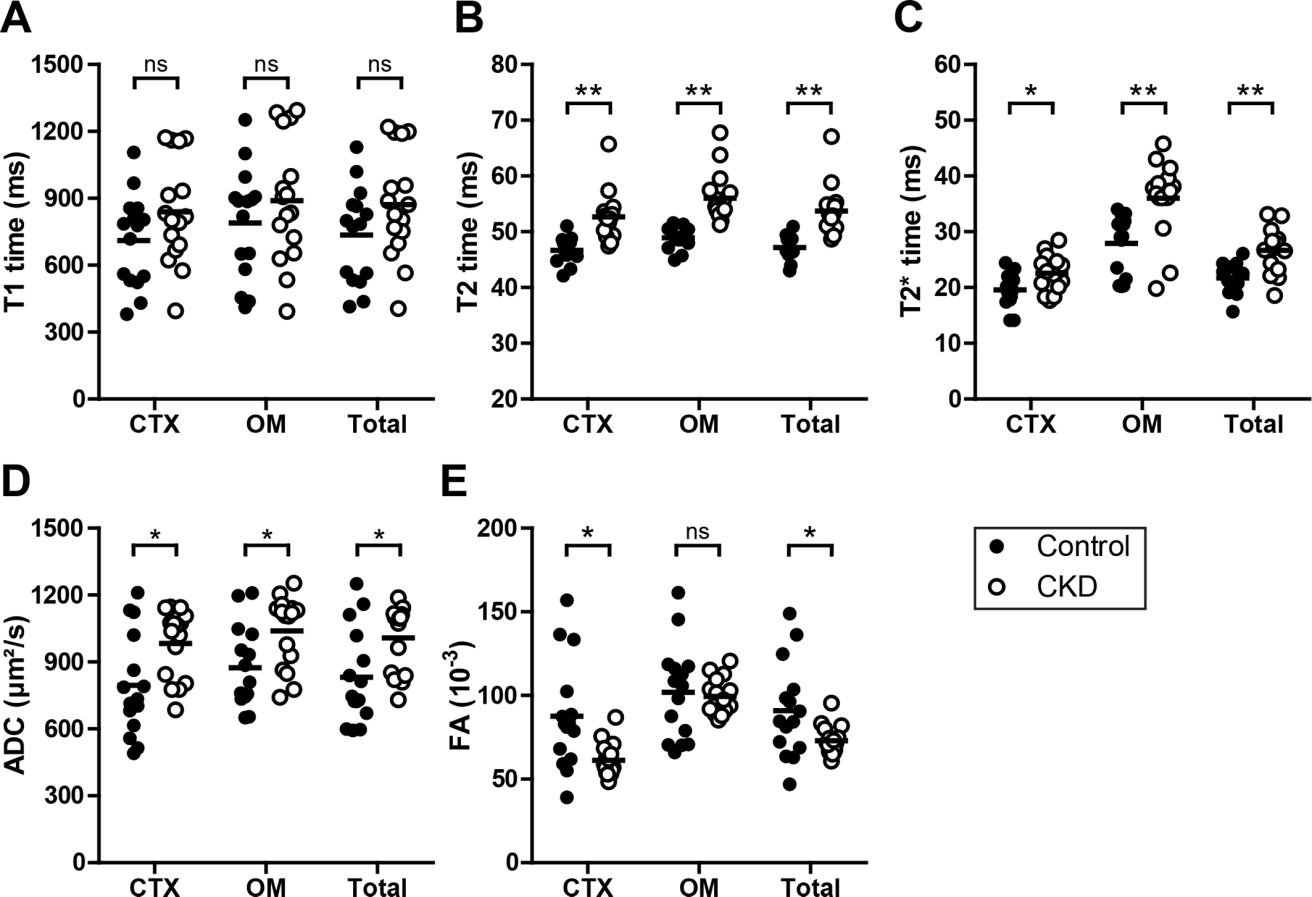
MRI parameters in control and CKD mice. T1 time (A), T2 time (B), T2* time (C), apparent diffusion coefficient (ADC, D), and fractional anisotropy (FA, E) were determined in the renal cortex (CTX), outer medulla (OM) and total volume (Total) of control (black circles, n = 15) and CKD kidneys (white circles, n = 16). Mean values are displayed by horizontal lines. * p <0.05, ** p <0.01.

T2, T2* time and ADC values were significantly elevated in total kidney volumes and specifically in cortical as well as outer medullary compartments of mice with CKD in comparison to control mice (Fig 1B–1D). Particularly, T2 and T2* values showed less variance than ADC values assessed by their interquartile range. In control and CKD mice T2 and T2* times were significantly higher in the outer medulla than in the cortex. The highest T2 and T2* times were found in the outer medulla of kidneys with CKD. In contrast, T1 time did not differ between groups (Fig 1A), while FA values significantly decreased in the cortex and total kidney volume of CKD in comparison to control mice (Fig 1E).

**Fig 2:**
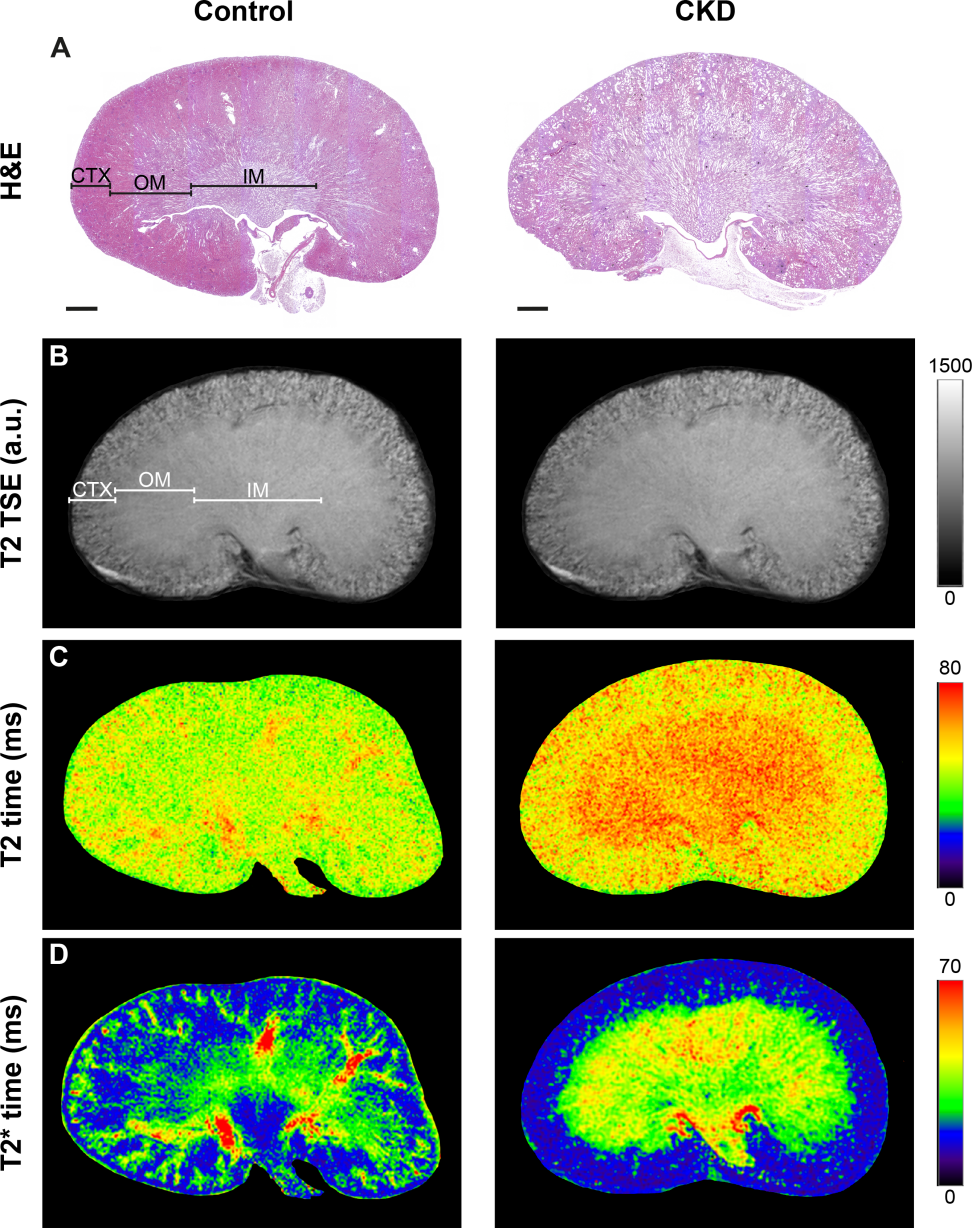
Comparison of histology, morphological and functional MRI. Representative hematoxylin & eosin (H&E) stained kidney sections (A), T2 weighted turbo spin echo (TSE) MR images (B), T2 relaxation time maps (C) and T2* relaxation time maps (D) of control (left) and CKD mice (right). Renal regions are indicated as CTX, cortex, OM, outer medulla, and IM, inner medulla. Scale bars, 1 mm.

In Fig 2 representative H&E stained histological sections (Fig 2A) are compared with T2-weighted morphological MR images (Fig 2B) and MRI voxel-based relaxation time maps for T2 and T2* times in pseudo-colors (Fig 2C and 2D). Morphological MR and histological images revealed that the regular structural organization of the kidneys was altered in CKD mice with a thinned, inhomogeneous renal cortex and seamless transition between outer and inner medulla in comparison to control kidneys (Fig 2A and 2B).

### Histological analyses in control and CKD mice

**Fig 3:**
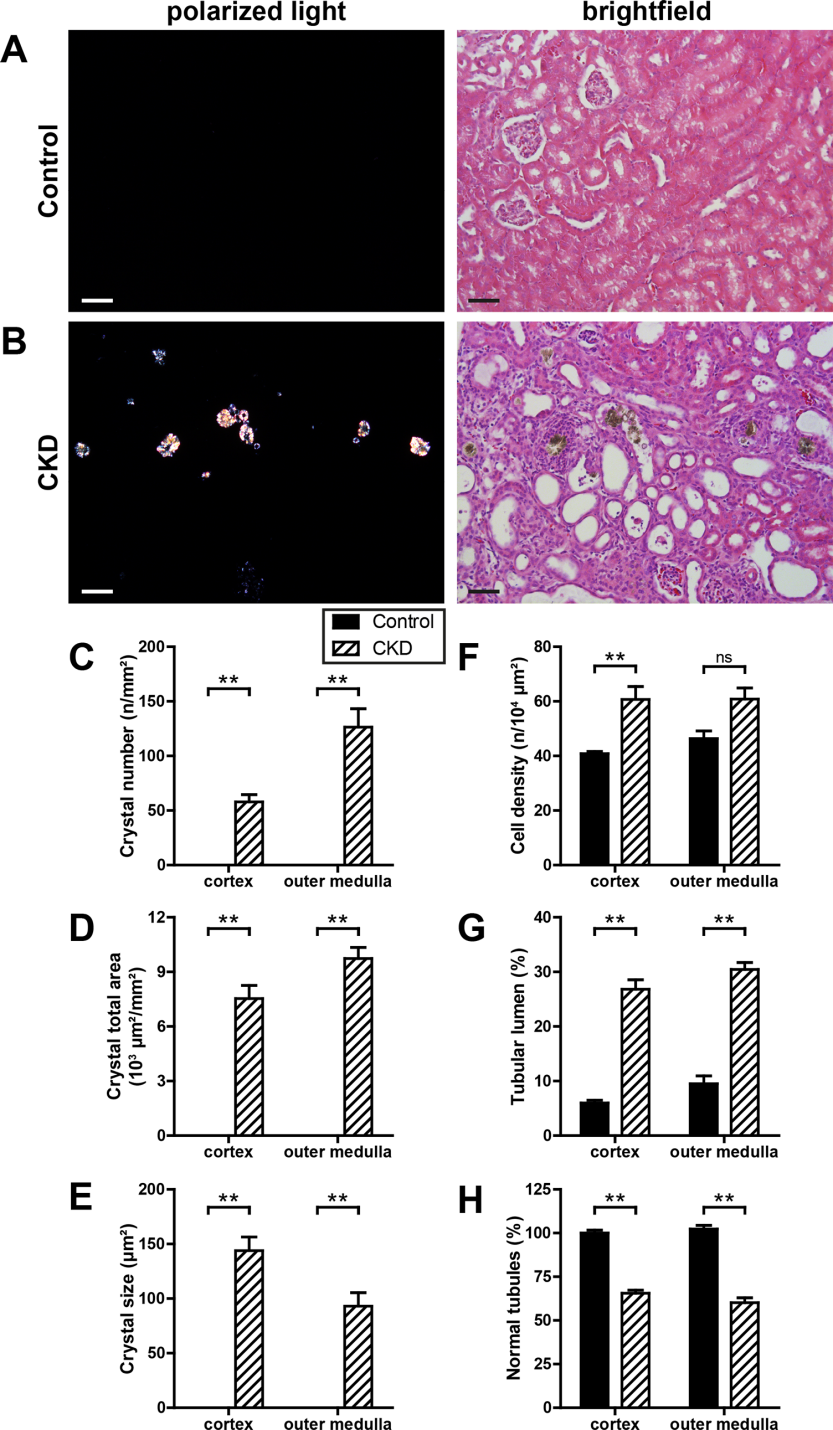
Kidney histomorphology. Representative hematoxylin & eosin (H&E) stained kidney sections from control (A) and CKD (B) mice under polarized light (left) and brightfield illumination (right). Number (C), total area (D) and size (E) of crystal deposits, cell density (F), as well as area of tubular lumina (G) and normal tubules (H) were quantified in the renal cortex and outer medulla of control (black bars, n = 15) and CKD kidneys (dashed bars, n = 16). Scale bars, 50 μm. ** p <0.01.

After MRI analysis paraformaldehyde-fixed kidneys were processed for histological assessment. In mice with adenine-induced CKD extensive deposition of dihydroxyadenine crystals in renal tubules and interstitium within the cortex and outer medulla were observed (Fig 3A–3E). Crystals were found more frequently in the outer medulla than in the cortex (Fig 3C and 3D). However, crystals in the outer medulla were smaller than in the cortex (Fig 3E). Crystal deposition resulted in significant cell infiltration of the interstitium, measured as cell density (Fig 3F), and dilatation of tubular lumina (Fig 3G). Hence, in CKD kidneys the remaining area of normal renal tubules was significantly reduced in comparison to control kidneys (Fig 3H).

**Fig 4:**
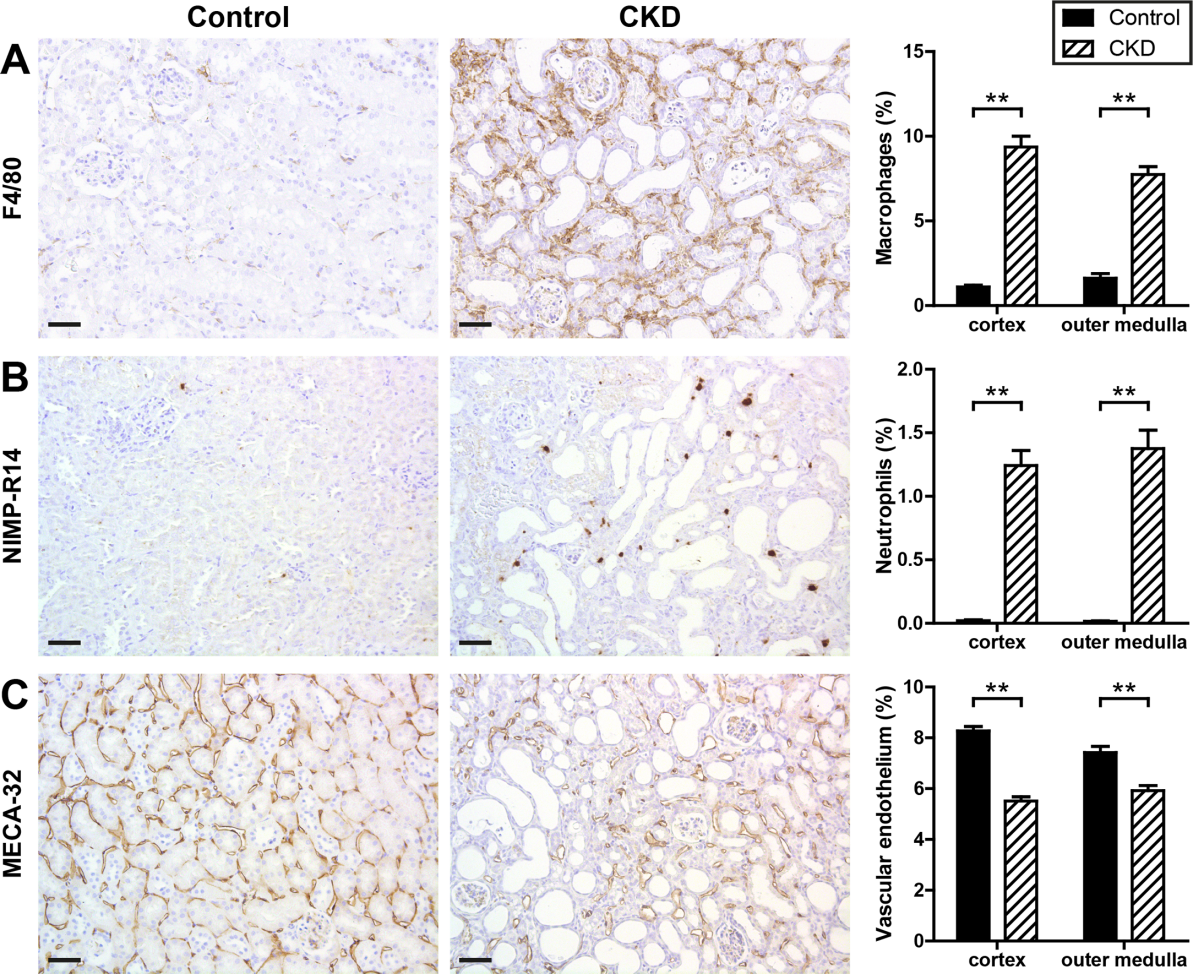
Tubulointerstitial inflammation and loss of peritubular capillaries in CKD kidneys. Kidney sections of control and CKD mice were stained for macrophages (A), neutrophils (B), and vascular endothelium (C). Shown are representative immunohistochemical stainings of control and CKD kidneys and quantitative analyses of each staining categorized for renal cortex and outer medulla. Black bars represent control (n = 15) and dashed bars CKD kidneys (n = 16). Scale bars, 50 μm. ** p <0.01.

Immunohistochemical characterization of the cellular infiltrate revealed that the tubulointerstitium in the renal cortex and outer medulla of CKD mice was extensively infiltrated with F4/80+ macrophages (Fig 4A) and moderately with NIMP-R14+ neutrophils (Fig 4B). Furthermore, in CKD kidneys peritubular capillaries were rarefied in comparison to healthy controls (Fig 4C).

**Fig 5:**
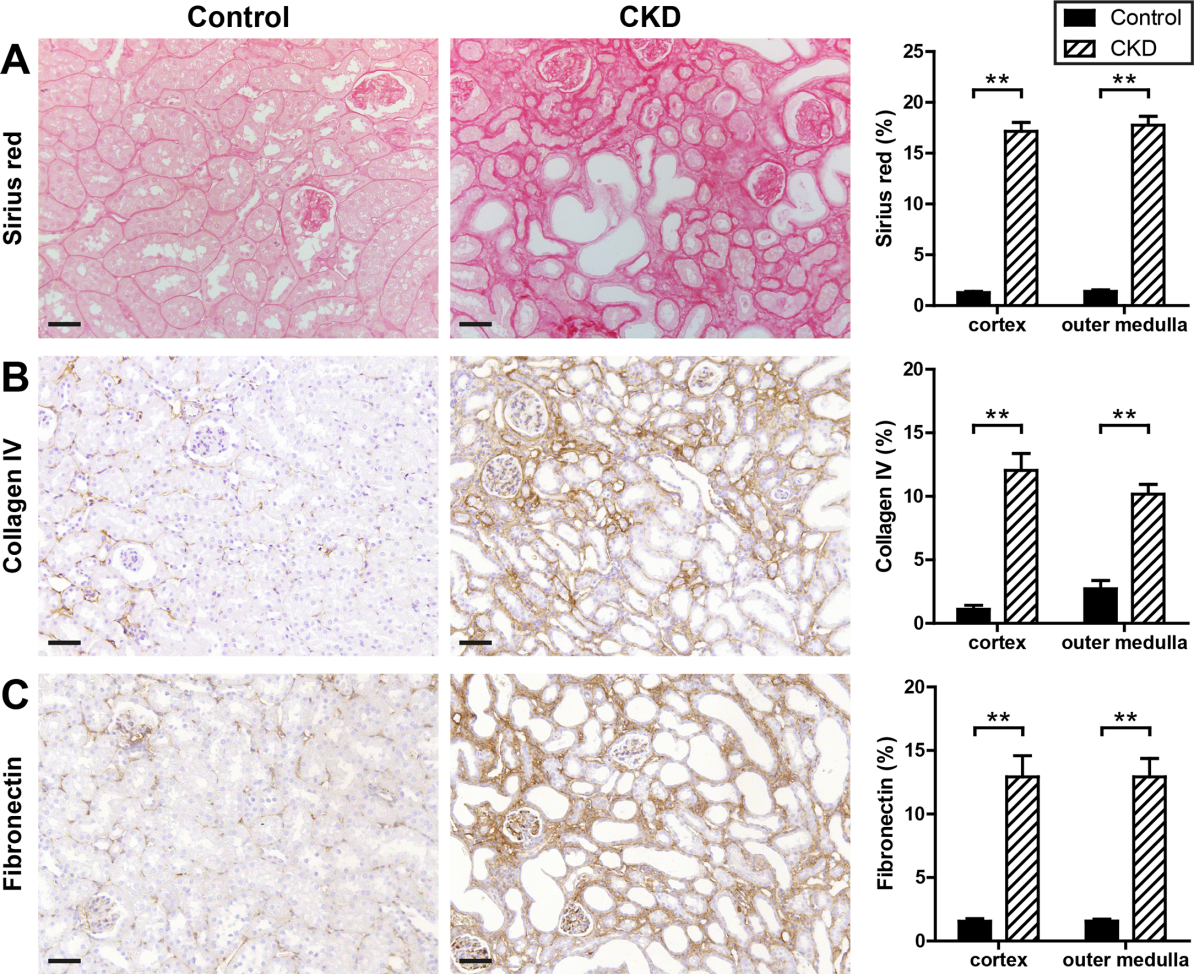
Interstitial fibrosis in CKD kidneys. Kidney sections of control and CKD mice were stained for Sirius red (A), collagen IV (B), and fibronectin (C). Shown are representative (immuno-) histological stainings of control and CKD kidneys and quantitative analyses of each staining categorized for renal cortex and outer medulla. Black bars represent control (n = 15) and dashed bars CKD kidneys (n = 16). Scale bars, 50 μm. ** p <0.01.

Tubulointerstitial inflammation in CKD kidneys was accompanied by interstitial fibrosis as assessed by Sirius red staining (Fig 5A), collagen type IV (Fig 5B), and fibronectin (Fig 5C) immunostaining.

### Correlation of histological and MRI parameters

Then we tested for potential associations between histological and MRI parameters. Pearson correlation revealed significant correlations of T2 time, T2* time, apparent diffusion coefficients, and fractional anisotropy with histological parameters (Table 1). In particular, T2 time correlated strongly or very strongly with deposition of extracellular matrix (as detected by Sirius red, collagen IV, and fibronectin stainings) and crystals, macrophage and neutrophil infiltration, tubular dilation as well as loss of peritubular capillaries and normal renal tubules. Furthermore, moderate correlations of T2* time and/or ADC with crystal deposition, fibrosis (albeit only with Sirius red staining), tubular dilation, neutrophil infiltration, and loss of renal microvessels and normal tubules were observed. We found only weak correlations between fractional anisotropy and histological parameters and no statistically significant correlations for T1 time.

**Table 1:** Correlation between MRI and histological parameters in the renal cortex and outer medulla of control and CKD kidneys.

	Collagen IV	Sirius red	Fibronectin	Vascular endothelium	Cell density	Macrophages	Neutrophils	Normal tubules	Tubular lumen	Crystal number	Crystal total area
T1 time	0.11	0.13	0.01	-0.19	-0.23	0.02	0.06	-0.19	0.01	-0.04	0.13
T2 time	0.85**	0.88**	0.83**	-0.89**	0.55**	0.82**	0.80**	-0.85**	0.80**	0.75**	0.85**
T2* time	0.23	0.41*	0.26	-0.38*	0.31	0.26	0.48**	-0.45**	0.46**	0.51**	0.48**
ADC	0.32	0.47**	0.37*	-0.40*	0.16	0.34*	0.52**	-0.59**	0.42*	0.50**	0.52**
FA	-0.30	-0.28	-0.28	0.32	-0.10	-0.37*	-0.27	0.38*	-0.19	-0.02	-0.20

ADC, apparent diffusion coefficient; FA, fractional anisotropy.

Data are Pearson’s correlation coefficient *r*.

* p <0.05.

** p <0.01.

We were also interested in the correlation of histopathological parameters of CKD with each other (Table 2). All histological parameters showed at least a moderate correlation. Particularly parameters related to fibrosis (Sirius red, collagen IV, and fibronectin) showed excellent correlation with macrophage infiltration, crystal deposition, and loss of normal renal tubules.

**Table 2:** Correlation of histological parameters among each other in the renal cortex and outer medulla of control and CKD kidneys.

	Collagen IV	Sirius red	Fibronectin	Vascular endothelium	Cell density	Macrophages	Neutrophils	Normal tubules	Tubular lumen	Crystal number	Crystal total area
Collagen IV	1.00	0.89**	0.93**	-0.85**	0.42*	0.91**	0.78**	-0.81**	0.73**	0.59**	0.78**
Sirius red	0.89**	1.00	0.89**	-0.89**	0.55**	0.93**	0.89**	-0.95**	0.87**	0.75**	0.93**
Fibronectin	0.93**	0.89**	1.00	-0.79**	0.51**	0.90**	0.80**	-0.79**	0.74**	0.71**	0.79**
Vascular endothelium	-0.85**	-0.89**	-0.79**	1.00	-0.52**	-0.86**	-0.76**	0.82**	-0.76**	-0.63**	-0.81**
Cell density	0.42*	0.55**	0.51**	-0.52**	1.00	0.57**	0.59**	-0.55**	0.71**	0.50**	0.43*
Macrophages	0.91**	0.93**	0.90**	-0.86**	0.57**	1.00	0.87**	-0.86**	0.84**	0.72**	0.86**
Neutrophils	0.78**	0.89**	0.80**	-0.76**	0.59**	0.87**	1.00	-0.89**	0.87**	0.75**	0.90**
Normal tubules	-0.81**	-0.95**	-0.79**	0.82**	-0.55**	-0.86**	-0.89**	1.00	-0.87**	-0.75**	-0.91**
Tubular lumen	0.73**	0.87**	0.74**	-0.76**	0.71**	0.84**	0.87**	-0.87**	1.00	0.75**	0.85**
Crystal number	0.59**	0.75**	0.71**	-0.63**	0.50**	0.72**	0.75**	-0.75**	0.75**	1.00	0.83**
Crystal total area	0.78**	0.93**	0.79**	-0.81**	0.43*	0.86**	0.90**	-0.91**	0.85**	0.83**	1.00

Data are Pearson’s correlation coefficient *r*.

* p <0.05.

** p <0.01.

Recently Farris et al. demonstrated the interrelatedness of tubulointerstitial injury in cortex and medulla of human kidney biopsies [26]. Thus, we also analyzed the correlation of MRI and histological parameters between renal compartments. Both MRI and histological parameters were strongly or very strongly correlated between renal cortex and outer medulla (Table 3).

**Table 3:** Correlation of MRI and histological parameters between renal cortex and outer medulla in control and CKD kidneys.

Parameter	Pearson‘s *r*
T1 time	0.98**
T2 time	0.93**
T2* time	0.70**
ADC	0.94**
FA	0.70**
Collagen IV	0.95**
Sirius red	0.99**
Fibronectin	0.96**
Vascular endothelium	0.88**
Cell density	0.74**
Macrophages	0.97**
Neutrophils	0.82**
Normal tubules	0.97**
Tubular lumen	0.93**
Crystal number	0.85**
Crystal total area	0.94**

ADC, apparent diffusion coefficient; FA, fractional anisotropy.

Data are Pearson’s correlation coefficient *r*.

** p <0.01.

### Effects of paraformaldehyde fixation on MRI parameters

**Fig 6:**
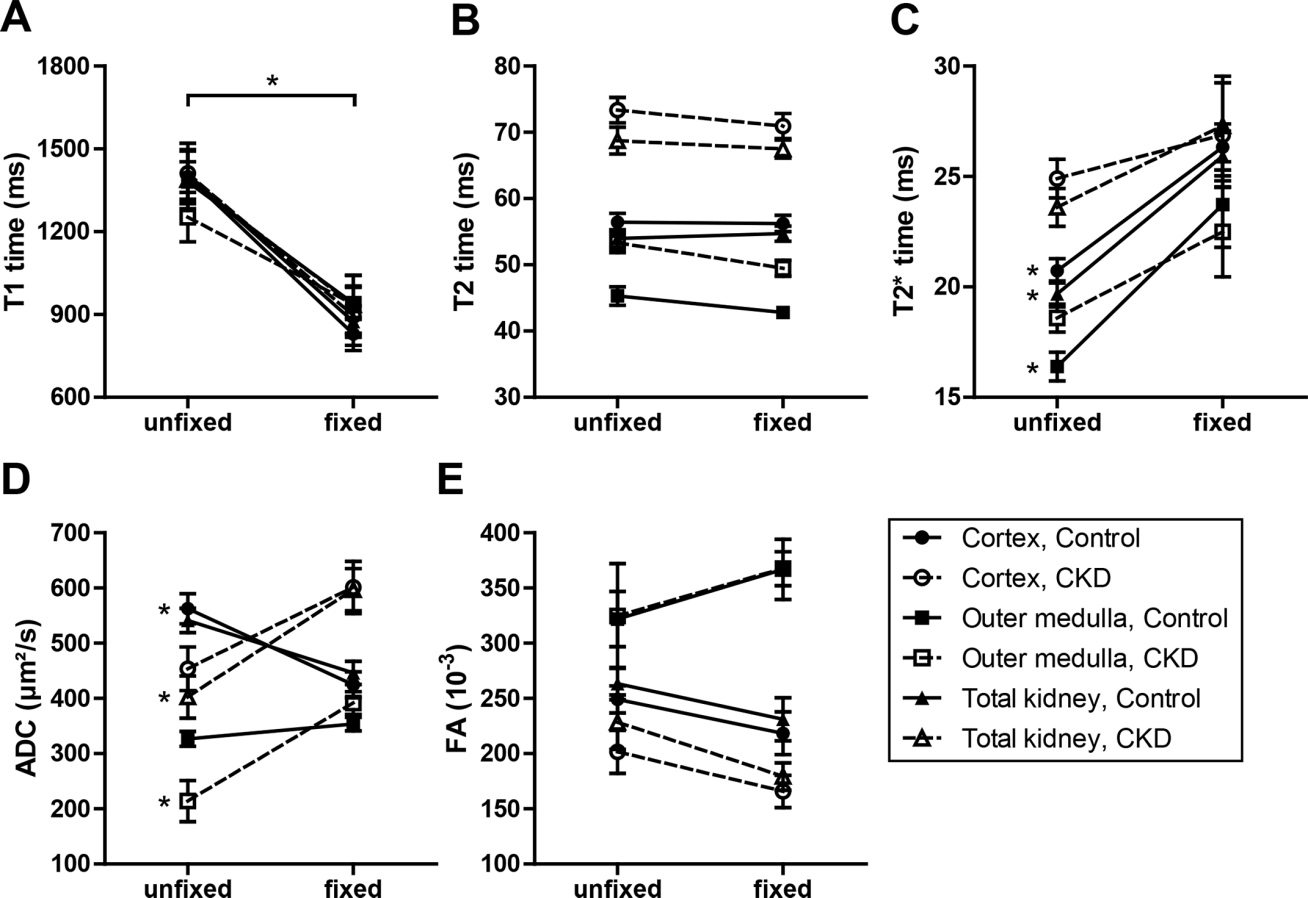
MRI parameters depending on paraformaldehyde fixation. T1 time (A), T2 time (B), T2* time (C), apparent diffusion coefficient (ADC, D) and fractional anisotropy (FA, E) were determined in the renal cortex, outer medulla and total volume of control (black symbols and solid lines, n = 8) and CKD (white symbols and dashed lines, n = 8) kidneys after extraction (unfixed) and 16 hours after fixation with paraformaldehyde. Given are mean and standard error. * p <0.05.

With regard to *in vivo* MRI measurements we investigated the potential influence of paraformaldehyde fixation on MRI-derived parameters by comparing MRI parameters of kidneys examined directly after nephrectomy and 16 hours after fixation with paraformaldehyde (Fig 6).

T2 times and fractional anisotropy were not influenced by paraformaldehyde fixation (Fig 6B and 6E). T1 times were significantly reduced in all kidney regions of both control and CKD mice after paraformaldehyde fixation (Fig 6A). T2* times increased due to fixation with paraformaldehyde. This effect was slightly higher in control mice reaching statistical significance, whereas T2* times obtained in CKD kidneys were statistically not different between unfixed and fixed samples (Fig 6C). Paraformaldehyde fixation increased apparent diffusion coefficients determined in CKD kidneys, which were statistically higher in the outer medulla and total volume of fixed in comparison to unfixed CKD kidneys, whereas in the cortex of control mice ADC values declined following fixation (Fig 6D).

Comparing MRI data acquired in fixed (Fig 1) and unfixed (S1 Fig) kidney samples revealed qualitatively similar changes for T1, T2, and T2* relaxation times as well as for fractional anisotropy between control and CKD kidneys in fixed and unfixed tissue specimens. Only changes of apparent diffusion coefficients differed in fixed and unfixed kidneys: while ADC values were lower in CKD than in control kidneys in unfixed tissue, they were higher in CKD than in control kidneys after fixation with paraformaldehyde.

## Discussion

Tubulointerstitial injury is both a key histopathological feature and an important predictor of the progression of renal dysfunction in CKD. Thus, determining the extent of tubulointerstitial changes without the need for kidney biopsy is of major clinical interest. Aiming to define MRI parameters for the assessment of tubulointerstitial injury, this study presents a comprehensive multiparametric MRI approach along with histopathological correlations in a murine model of adenine-induced nephropathy. As contrast agent application remains critical in patients with reduced renal function [27], the presented parameters were obtained using techniques not requiring gadolinium-containing contrast agents.

We demonstrate significant differences between control and CKD kidneys regarding T2 and T2* relaxation times as well as the apparent diffusion coefficient. In particular, T2 relaxation time was significantly increased in CKD kidneys and correlated very well with the extent of fibrosis and inflammatory cell infiltrates in the renal cortex and medulla, rendering this parameter a promising candidate for quantitative assessment of tubulointerstitial alterations in CKD by MRI parameter mapping. Changes in T2 relaxation times and their association with inflammatory cell infiltration and interstitial fibrosis have been reported in rodent models of ischemia-reperfusion injury [14], allograft nephropathy [16,17], unilateral ureteral obstruction [28] as well as oxalate-induced kidney disease [29]. However, these studies lacked the detailed quantitative correlation analysis of MRI and histological parameters provided in our study.

In contrast, T2* time values showed greater variability and weaker correlations with histological parameters than T2 time values.

For diffusion-weighted imaging of the kidney, previous experimental studies reported reduced apparent diffusion coefficient and fractional anisotropy values in rodent models of ischemia-reperfusion [14,30], diabetic nephropathy [31–33], allograft nephropathy [16,17], and ureteral obstruction [34–38]. When correlated with histology, apparent diffusion coefficient and fractional anisotropy levels were found to decrease with progressive renal fibrosis [16,32,34,35,37,38]. These findings were confirmed in clinical studies of patients with CKD [39–41] or allograft nephropathy after kidney transplantation [42–46], in which apparent diffusion coefficient and fractional anisotropy values correlated positively with renal function and inversely with tubulointerstitial injury. Diffusion-weighted MR imaging simultaneously provides information on diffusion and perfusion. However, it is a question of debate if both processes can be separated in the kidneys [39]. The reduction of the apparent diffusion coefficient in CKD has commonly been attributed to limited kidney perfusion (blood and/or tubular flow), cell swelling accompanied by a loss of cell membrane integrity and/or an increase in tissue cellularity restricting diffusion of water in the interstitial space [47,48]. In our *ex vivo* experimental setup perfusion artefacts were excluded. We found reduced apparent diffusion coefficients in unfixed CKD kidneys corroborating the results of previous *in vivo* studies, whereas in paraformaldehyde-fixed kidney samples with CKD apparent diffusion coefficients were elevated.

Although we also found significantly reduced fractional anisotropy values in the renal cortex of mice with adenine-induced nephropathy, we could not detect substantial correlations with histological parameters.

Changes in T1 relaxation times have been reported in rodent models of ischemic AKI [15], allograft rejection [16], ureteral obstruction [38] and a rabbit model of anti-glomerular basement membrane disease [49]. In our study, no significant changes of T1 values were detected between control and CKD mice.

The high-resolution MRI scans within this study were performed on paraformaldehyde-fixed kidneys to avoid motion and flow artefacts that may hamper *in vivo* measurements. By cross-linking protein amine groups via methylene bridges, tissues are kept metabolically and structurally stable after fixation [50]. In contrast to other tissue processing procedures (alcohol dehydration, clearing in xylene, and infiltration with paraffin), fixation with formaldehyde itself causes only little volume changes [51]. However, formaldehyde fixatives may significantly alter water diffusivity in tissue and thus affect diffusion properties as demonstrated for brain and other organs [52]. Shepherd et al. observed significant reductions of T1 and T2 times as well as a significant increase of apparent diffusion coefficients after aldehyde fixation of rat brain cortex slices. Interestingly, at least T2 reductions were completely reversible by gentle washing with phosphate-buffered saline [52]. We also routinely wash paraformaldehyde-fixed tissue samples with phosphate-buffered saline to remove excess free fixative solution before further histological processing. For kidneys the effect of paraformaldehyde fixation on MRI parameters has not yet been examined. Here we observed that both T2 relaxation time and fractional anisotropy were not influenced by fixation with paraformaldehyde for 16 h followed by washing with phosphate-buffered saline. In contrast, T1 times significantly decreased in fixed kidney samples, whereas T2* times and the apparent diffusion coefficient were significantly elevated (at least partially, depending on the region of interest) in accordance with the findings in brain. Therefore, T1 and T2* times as well as apparent diffusion coefficients have to be interpreted cautiously in paraformaldehyde-fixed kidney samples in both CKD and control kidneys, whereas T2 times and FA values were not altered by paraformaldehyde fixation.

Prolonged chronic injurious stimuli to kidney tissue may result in tubulointerstitial fibrosis which is characterized by accumulation of extracellular matrix components, activation and expansion of (myo-) fibroblasts, infiltration of inflammatory cells, tubular atrophy, and microvascular rarefaction [5,6]. We found very close correlations between histopathological determinants of CKD in our study. Parameters of fibrosis were highly correlated with macrophage infiltration and loss of renal microvessels and normal tubules. However, this might make it difficult to deduce which individual histopathological characteristic is reflected by changes in T2 relaxations time.

Moreover, histological and radiological parameters were closely correlated between renal compartments indicating that (outer) medullary and cortical tissue injuries are interrelated which might have potential theoretical and practical implications. First, there is extensive literature and knowledge about histopathological alterations of renal cortical tissue in a variety of kidney diseases, whereas the renal medulla is often neglected mainly because human renal biopsies mostly do not include the medulla. Our study and the observations by Farris et al. [26] might indicate that cortical tissue damage might be reflected in the medulla or even vice versa. The latter concept might imply the existence of primary medullary kidney diseases that spread into the renal cortex and being mistaken as primary cortical diseases. Second, MRI analysis of kidney tissue samples can be performed in either renal cortex or medulla, however combinations should be avoided.

Due to technical limitations it was not possible to exactly match the regions of histological and radiological evaluation. Nevertheless, we found very close correlations between histopathological and radiological analyses. Correlations might even increase, if both techniques could be applied to the exact same region of interest.

A major strength of our study is the detailed assessment of quantitative MRI parameters in CKD kidneys without motion artefacts and their correlation with quantitative histological findings. Our data demonstrate a strong potential of MRI parameters to determine renal tissue characteristics. Particularly T2 relaxation time is a promising MRI parameter to quantitatively assess renal fibrosis, as it showed the best correlation with histopathological characteristics of kidney fibrosis and remained unaffected by paraformaldehyde fixation. Therefore, it could be a robust parameter for the use in patients as well. Adding a sequence for quantification of T2 relaxation times in a renal MRI scan is fully compatible with clinical routine protocols and thus cost-effective as the additional examination time is in the range of few minutes only.

As *ex vivo* MRI measurements reflect the histologic properties of kidneys tissue architecture as accurately as possible, radiological and histopathological analysis of human kidney biopsies might be a next step to high-resolution *in vivo* imaging of human CKD kidneys. In the long term, multiparametric MRI could potentially substitute renal biopsies for diagnosis and evaluation of CKD, thus preserving already impaired renal tissue from additional iatrogenic damage.

## Supporting information

S1 Fig MRI parameters in unfixed control and CKD kidneys. Relaxation times T1 (A), T2 (B), and T2* time (C), as well as the diffusion parameters apparent diffusion coefficient (ADC, D) and fractional anisotropy (FA, E) were determined in the renal cortex (CTX), outer medulla (OM) and total volume (Total) of control (black circles, n = 6–8) and CKD kidneys (white circles, n = 6–8) directly after nephrectomy. Mean values are displayed by horizontal lines. * p <0.05, ** p <0.01.
(TIF)
