# Correction: Multiparametric magnetic resonance imaging of experimental chronic kidney disease: A quantitative correlation study with histology

**DOI:** 10.1371/journal.pone.0218876

**Published:** 2019-06-19

**Authors:** Gunnar Schley, Jutta Jordan, Stephan Ellmann, Seymour Rosen, Kai-Uwe Eckardt, Michael Uder, Carsten Willam, Tobias Bäuerle

Fig 2 is incorrect. Please see the correct Fig 2 here.

**Fig 2:**
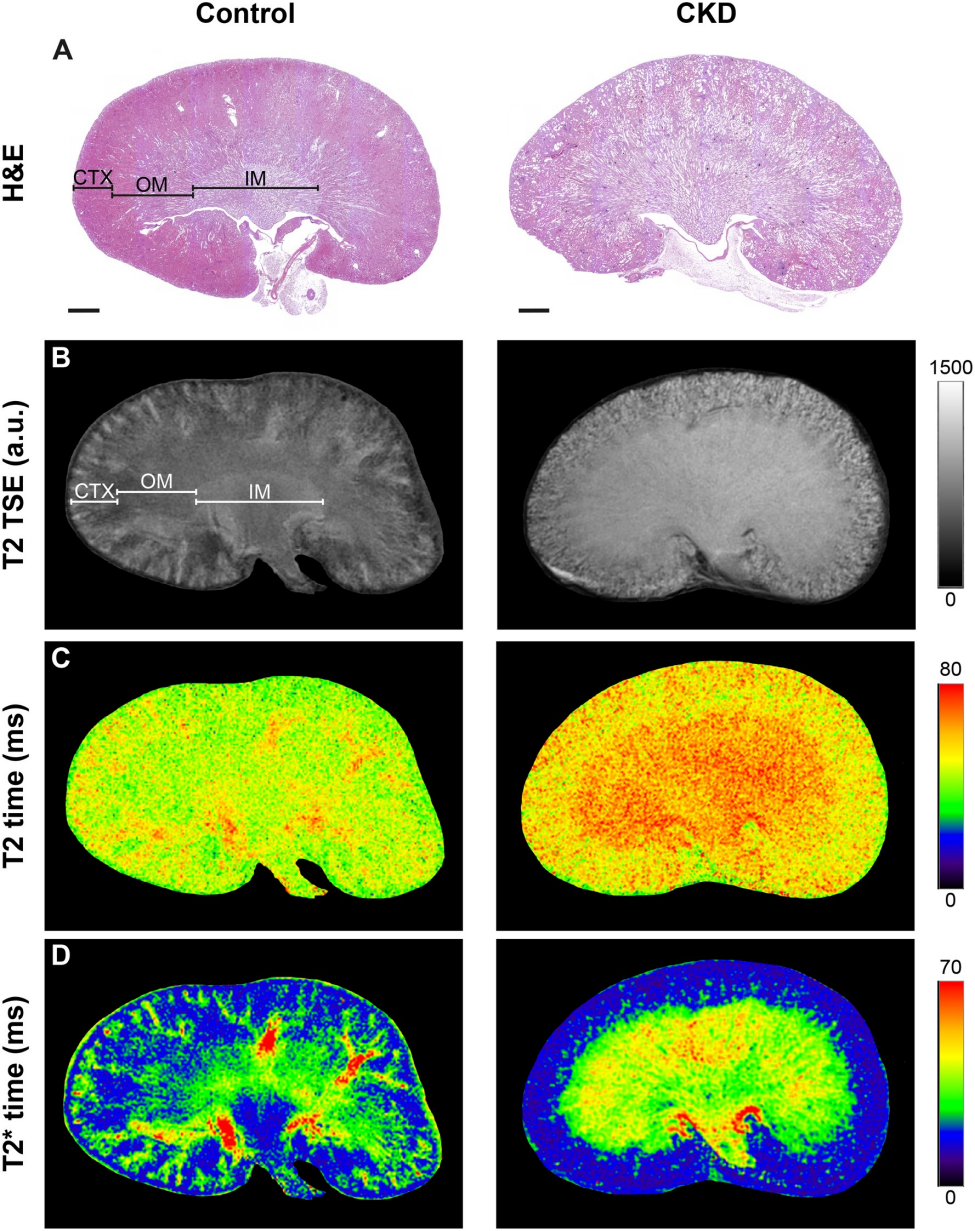
Comparison of histology, morphological and functional MRI. Representative hematoxylin & eosin (H&E) stained kidney sections (A), T2 weighted turbo spin echo (TSE) MR images (B), T2 relaxation time maps (C) and T2* relaxation time maps (D) of control (left) and CKD mice (right). Renal regions are indicated as CTX, cortex, OM, outer medulla, and IM, inner medulla. Scale bars, 1 mm.
