# Supplementary figures and images for: Multiparametric magnetic resonance imaging of experimental chronic kidney disease: A quantitative correlation study with histology
Source: PLoS One. 2018 Jul 16;13(7):e0200259. doi: 10.1371/journal.pone.0200259 (PMC6047786; doi:10.1371/journal.pone.0200259)

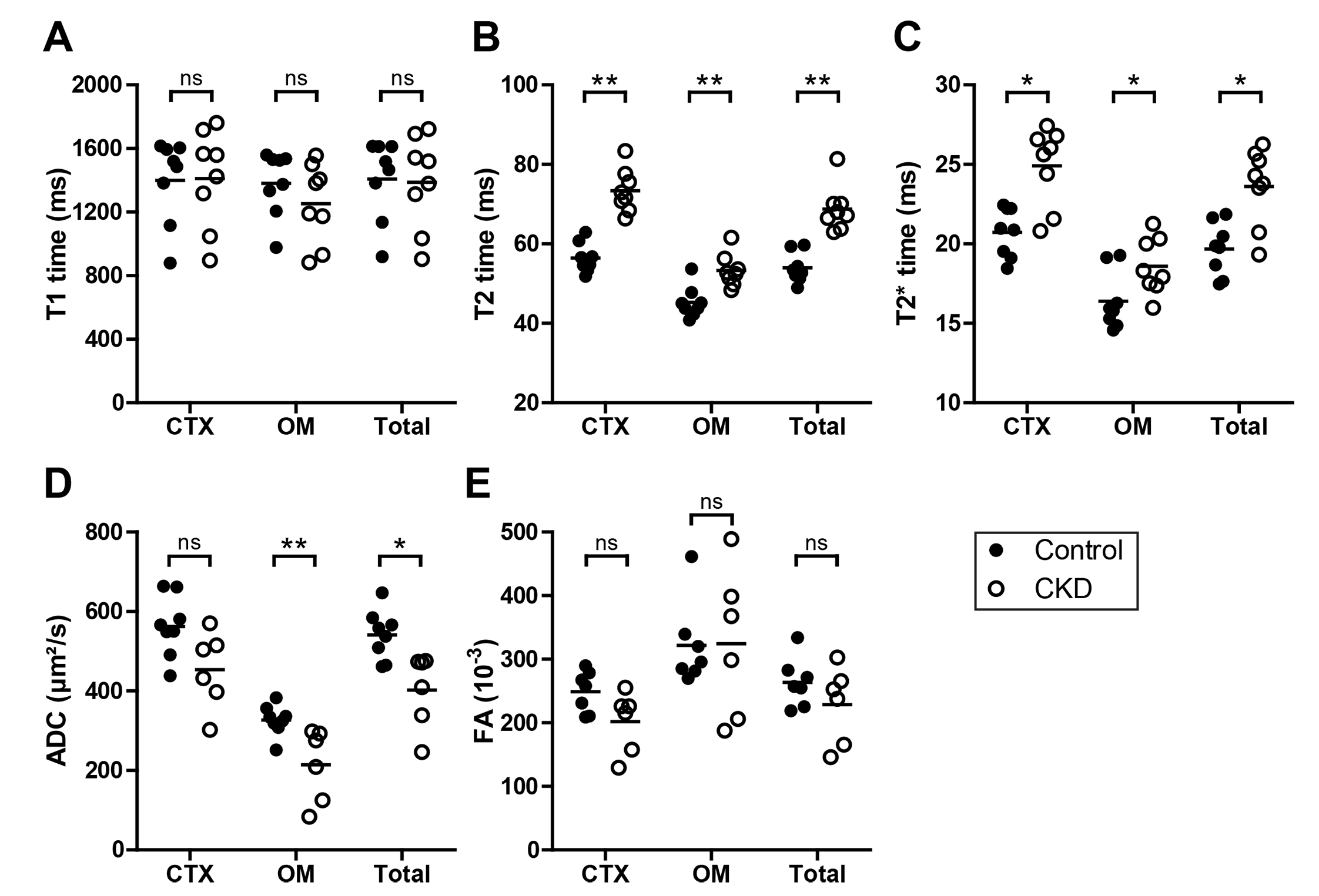

Supplement: S1 Fig — Relaxation times T1 (A), T2 (B), and T2* time (C), as well as the diffusion parameters apparent diffusion coefficient (ADC, D) and fractional anisotropy (FA, E) were determined in the renal cortex (CTX), outer medulla (OM) and total volume (Total) of control (black circles, n = 6–8) and CKD kidneys (white circles, n = 6–8) directly after nephrectomy. Mean values are displayed by horizontal lines. * p <0.05, ** p <0.01. (TIF) [file pone.0200259.s001.tif]
